# 

**Published:** 1894-07

**Authors:** 


					﻿C8TABLISHE0 1855.	SUBSCRIPTION,	|
ATLANTA
JVIEDIG Ah AJW SURGIGflli
JOURNAL.
OLD SERIES, VOL. XXVI..,	.	T	. n_ . „ _
NEW SERIES, VOL. XI. (	ATLANTA, Ga., JULY, 189-1. ISO.
LUTHER B. GRANDY, M.D.,	M. B. HUTCHINS, M.D.,
MANAGING "EDITOR.	BUSINESS MANAGER.
				

